# Manufacture of Micromirror Arrays Using a CMOS-MEMS Technique

**DOI:** 10.3390/s90806219

**Published:** 2009-08-06

**Authors:** Pin-Hsu Kao, Ching-Liang Dai, Cheng-Chih Hsu, Chyan-Chyi Wu

**Affiliations:** 1 Department of Mechanical Engineering, National Chung Hsing University, Taichung, 402 Taiwan; E-Mail: d9461402@mail.nchu.edu.tw (P.-H.K.); 2 Department of Electro-Optical Engineering, Yuan Ze University, Taoyuan, 320 Taiwan; E-Mail: cchsu@saturn.yzu.edu.tw (C.-C.H.); 3 Department of Mechanical and Electro-Mechanical Engineering, Tamkang University, Tamsui, 251 Taiwan; E-Mail: ccwu@mail.tku.edu.tw (C.-C.W.)

**Keywords:** micromirror array, microactuator, CMOS-MEMS

## Abstract

In this study we used the commercial 0.35 μm CMOS (complementary metal oxide semiconductor) process and simple maskless post-processing to fabricate an array of micromirrors exhibiting high natural frequency. The micromirrors were manufactured from aluminum; the sacrificial layer was silicon dioxide. Because we fabricated the micromirror arrays using the standard CMOS process, they have the potential to be integrated with circuitry on a chip. For post-processing we used an etchant to remove the sacrificial layer and thereby suspend the micromirrors. The micromirror array contained a circular membrane and four fixed beams set symmetrically around and below the circular mirror; these four fan-shaped electrodes controlled the tilting of the micromirror. A MEMS (microelectromechanical system) motion analysis system and a confocal 3D-surface topography were used to characterize the properties and configuration of the micromirror array. Each micromirror could be rotated in four independent directions. Experimentally, we found that the micromirror had a tilting angle of about 2.55° when applying a driving voltage of 40 V. The natural frequency of the micromirrors was 59.1 kHz.

## 1. Introduction

The popularity of portable computers, mobile communication devices, and personal electronics is growing rapidly, with many consumers desiring bright, high-resolution, large-viewing-area, and compact displays. The main challenges when fabricating portable displays and microvision systems are the size, power consumption, and the choice of manufacturing process. Microelectromechanical systems (MEMS) technology, mostly developed during the past two decades, can be used to overcome these challenges.

Micromirrors are among the important optical devices used in scanned display and image systems. They are also applied widely in projection display systems [1], optical scanners [2], optical waveguides [3], and optical switches [4,5], and for signal processing in rear-projection televisions [6]. Micromirrors allow scanners in point-to-point scanning to produce distortion-corrected images on highly curved surfaces. For example, Chiou and Lin [7] developed a torsion micromirror device possessing multiple driving electrodes to investigate the effect on the tilting angle of the arrangement of driving electrodes. Jang and Kim [8] presented a digitally operated micromirror array possessing a torsional spring; they studied the deviations in pull-in voltages obtained using different spring sizes, finding they were clearly affected by the mirror gap and the spring width. Several studies have revealed the feasibility of manufacturing micromirrors using surface or bulk micromachining processes. For instance, Al-Aribe and Knopf [9] proposed a MEMS torsion micromirror for use in optical switches. In general, however, MEMS-based micromirrors may possess some problematic features, such as high process complexity, high driving voltages, high power consumption, and difficulty in combining them with circuits on chips. Standard complementary metal oxide semiconductor (CMOS) processes allow the fabrication of small devices; the CMOS-MEMS technique is the name of the commercial CMOS process used to fabricate MEMS devices [10–13]. Micromirrors manufactured using the CMOS-MEMS technique have many advantageous properties, including stable processing, ready integration with circuits, and low cost.

Many actuation methods can be used on MEMS actuators, including thermoelectric, pressured, magnetic, and electrostatic force methods. Among these approaches, the electrostatic force method is the most popular for achieving rapid response times, low power consumption, and simple electronics. Many researchers have developed electrostatic actuating micromirrors, such as Texas Instruments’ digital micromirror device (DMD) [14], AT&T’s optical switches [15], and Cheng and co-worker’s mirror integrated with circuitry [16]. Herein, we discuss a rotatable micromirror array that is driven using electrostatic forces.

In this paper, we present a surface-micromachined and rotatable micromirror array fabricated using the commercial 0.35 μm CMOS process. The post-CMOS process in this work that requires only one maskless wet etching process is simpler than that of Cheng *et al.* [16]. Thereby, the post-process is easy to execute and low cost. Many electrostatically actuated micromirrors not only rotate but also displace downward when they are actuated. We introduce a center pin to prevent such downward displacement. The natural frequency of this micromirror array is suitable for commercial project displays in which the dot pitch is sufficiently small to provide high resolution.

## 2. Design and Simulation

**Figure 1.**
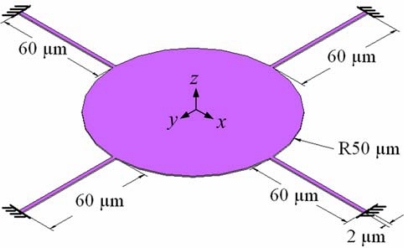
Schematic representation of the micromirror.

Figure 1 displays a schematic representation of a micromirror in our array. Material of the mirror is aluminum. Its structure is circular, suspended and supported by four fixed beams and one pin. The pin, located at the center of the micromirror, is a holder providing the tilt stability of the micromirror; its diameter is 0.5 μm. The diameter of the mirror is 100 μm; each supported beam is 60 μm long, 2 μm wide and 0.5 μm thick. Four quarter-circle fixed electrodes, which are the driving units used to tilt the mirror, are located below the suspended mirror; each fixed electrode controlled one tilting direction. The mirror is actuated by electrostatic force, the strength of which is given by:

(1) $$F=\frac{\varepsilon {\mathrm{AV}}^{2}}{2d^{2}}$$

where *ε* is the permittivity, *A* is the overlapping area, *d* is the distance of the parallel electrodes, and *V* is the driving voltage applied to the fixed electrode and the mirror.

When a dc bias is applied to the mirror and the fixed electrode, the mirror tilted toward the fixed electrode, allowing the mirror to be rotated in the *x* and *y* directions. Because the gap between the mirror and the driving electrode is 2.6 μm, the permittivity in air is 8.85 × 10^−12^ F/m, and the overlapping area is 1,590 μm^2^, Equation (1) suggests that the electrostatic force of 1.2759 μN would result under an applied driving voltage of 40 V.

**Figure 2.**
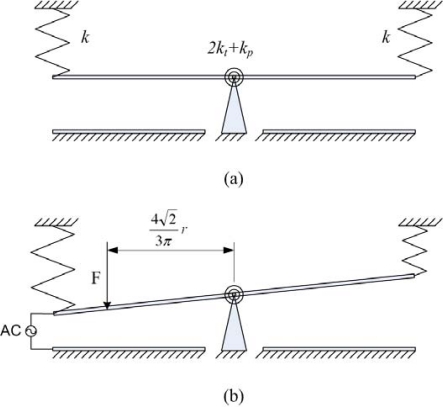
Dynamic model of a micromirror operated in the (a) absence and (b) presence of a driving voltage.

Figure 2(a) presents a model of the dynamic motion of a micromirror suspended and fixed by four cantilever beams. The micromirror is positioned flat and horizontal to the surface in the absence of a driving voltage. As shown in Figure 2(b), the micromirror is tilted upon applying a driving voltage. Among the four cantilever beams, two are transformed into springs (*k*) and the other two are equal to torsion springs (*k*_t_). If applying an ac voltage of *V*sin*ωt* to the micromirror, the micromirror produces a harmonic motion. The lower electrodes are quarter-circle shape, so their center of gravity are at the position of $\frac{4\sqrt{2}}{3\pi }r$. From Newton’s law, the equation of motion of the micromirror is given by:

(2) $$I_{m}\ddot{\theta }+c\dot{\theta }+\left(2{\mathrm{kr}}^{2}+2k_{t}+k_{p}\right)\theta =\frac{4\sqrt{2}}{3\pi }\;\mathrm{rF}\;\text{sin}\;\omega t$$

and:

(3) $$I_{m}=\frac{1}{4}\;{\mathrm{mr}}^{2}$$

(4) $$k=\frac{12\mathrm{EI}}{L^{3}}$$

(5) $$k_{t}=\frac{\mathrm{GJ}}{L}$$

(6) $$k_{p}=\frac{4{\mathrm{EI}}_{p}}{L_{p}}$$

where *θ* represents the rotated angle of the mirror when the driving voltage is applied; *θ̇* is the angular velocity; *θ̈* is the angular acceleration; and *F* is defined by Equation (1); *I_m_* is the mass moment of inertia of the mirror; *c* is the air damping; *r* is the radius of the mirror, *m* is the mass of the mirror; *E* is the Young’s modulus of the cantilever beams and the pin, *I* is the moment of inertia of the cantilever beams; *G* is the shear modulus of the torsion bars; *J* is the polar moment of inertia of the torsion bars; *L* is the length of the cantilever beams and the torsion bars; *k_p_* is the stiffness of the pin; *I_p_* is the moment of inertia of the pin; and *L_p_* is the length of the pin. Equation (2) can be written as:

(7) $$\ddot{\theta }+2\varsigma \omega_{n}\dot{\theta }+\omega_{n}\theta =M_{0}\;\text{sin}\;\omega t$$

and:

(8) $$k_{e}=2{\mathrm{kr}}^{2}+2k_{t}+k_{p}$$

(9) $$\varsigma =\frac{c}{2I_{m}\;\omega_{n}}$$

(10) $$\omega_{n}=\sqrt{\frac{k_{e}}{I_{m}}}$$

(11) $$M_{0}=\frac{4\sqrt{2}\mathrm{rF}}{3\pi I_{m}}$$

where *k_e_* represents the equivalent stiffness of the mirror; *ζ* is the damping ratio; *ω_n_* is the natural frequency of the mirror, and *M*_0_ is the moment. The particular solution of Equation (7) can be expressed as [17]:

(12) θ(t)=Θ sin(ωt−ϕ)

and:

(13) $$\Theta =\frac{\frac{M_{0}}{k_{e}}}{\sqrt{{\left(1-r_{\omega }^{2}\right)}^{2}+{\left(2\varsigma r_{\omega }\right)}^{2}}}$$

where Θ and *ϕ* are the amplitude and phase angle of the response, respectively; *r_ω_* is the frequency ratio and *r_ω_* = *ω*/*ω_n_*. The maximum amplitude occurs when $r_{\omega }=\sqrt{1-2\varsigma^{2}}$ [17] Substituting $r_{\omega }=\sqrt{1-2\varsigma^{2}}$ and Equations (1) and (11) into Equation (13), the maximum amplitude of the rotated angle in the micromirror can be obtained:

(14) $$\Theta_{\text{max}}=\frac{\sqrt{2}\varepsilon {\mathrm{rAV}}^{2}}{3\pi d^{2}\;k_{e}\varsigma \sqrt{1-\varsigma^{2}}}$$

**Figure 3.**
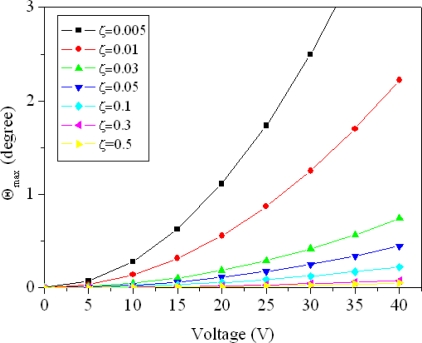
Maximum amplitude of the rotated angle in the micromirror at different damping ratios.

In the design, the equivalent stiffness of the mirror, *k_e_*, is about 1.35 × 10^−9^ N-m/rad, and the overlapping area of the electrodes, A, is 1,590 μm^2^. Figure 3 shows the maximum amplitude of the micromirror with different damping ratios, which is evaluated by Equation (14). In addition to the equivalent stiffness and the geometric shape of the mirror, the maximum rotated angle of the mirror depends on the driving voltage and the damping ratio.

The resonant frequency of the micromirror is given by:

(15) $$f=\frac{\omega_{n}}{2\pi }=\frac{1}{2\pi }\sqrt{\frac{k_{e}}{I_{m}}}$$

**Figure 4.**
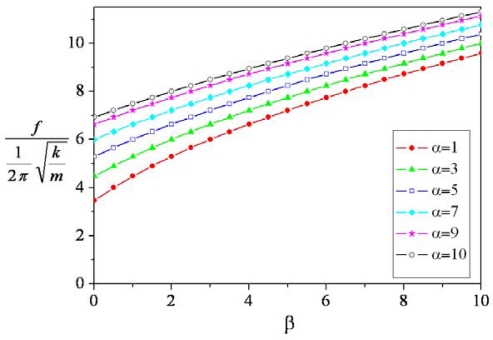
Dimensionless resonant frequency vs. stiffness ratio.

In accordance with Equation (15), we know that the resonant frequency of the mirror changes as the effective stiffness and the mass moment of inertia of the mirror vary. The equivalent stiffness in Equation (8) contains three factors of *k*, *k_t_* and *k_p_*. so it is not easy to determine the values of *k*, *k_t_* and *k_p_* according to the equivalent stiffness. In order to easier determine the values of *k*, *k_t_* and *k_p_*, the dimensionless resonant frequency is introduced. Substituting Equations (3) and (8) into Equation (15), the dimensionless resonant frequency of the micromirror can be obtained:

(16) $$\frac{f}{\frac{1}{2\pi }\sqrt{\frac{k}{m}}}=2\sqrt{2+\alpha +2\beta }$$

and:

(17) $$\alpha =\frac{k_{p}}{{\mathrm{kr}}^{2}}$$

(18) $$\beta =\frac{k_{t}}{{\mathrm{kr}}^{2}}$$

where *α* and *β* are the stiffness ratio. Figure 4 shows the dimensionless resonant frequency of the micromirror, which is computed by Equation (16). The results depict that the dimensionless resonant frequency of the micromirror are 4.47 and 10 corresponding to the stiffness ratios of 0 and 10, respectively, at *α* = 3.

In our design, the value of *E* is 70 GPa; *I* is 2.08 × 10^−26^ m^4^; *G* is 28 GPa; *L* is 60 μm; *I_p_* is 3.07 × 10^−27^ m^4^; *L_p_* is 1.4 μm and *J* is 3.54 × 10^−25^ m^4^. We substitute these values into Equations (4), (5) and (6), the stiffness of the cantilever beams, the torsion bars and the pin can be evaluated, and the results are *k* = 0.081 N/m, *k_t_* = 1.646 × 10^−10^ N-m/rad and *k_p_* = 6.136 × 10^−10^ N-m/rad. According to Equations (17) and (18) with *r* = 50 μm, we yield that the stiffness ratio of *α* and *β* are 3 and 0.81, respectively. As shown in Figure 4, the dimensionless resonant frequency is 5.14 at *α* = 3 and *β* = 0.81. The mass of the mirror is 1.158 × 10^−11^ kg, so we calculate the resonant frequency of the micromirror to be 68.7 kHz. All symbols are listed in Table 1, and the material and geometric properties in the micromirrors are summarized in Table 2.

## 3. Fabrication of the Micromirror Array

**Figure 5.**
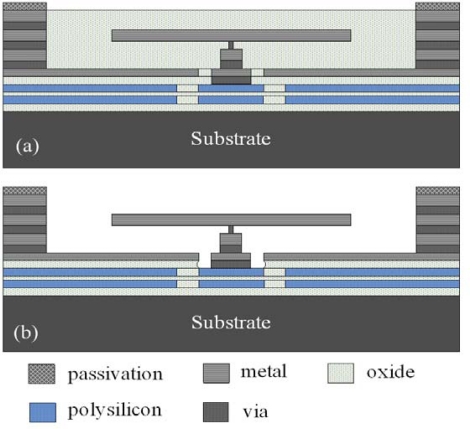
Process flow of the micromirror: (a) after completion of the CMOS process; (b) after completion of the post-process.

The micromirror array was manufactured using the commercial CMOS process of the Taiwan Semiconductor Manufacturing Company (TSMC), according to the micromirror layout defined in Figure 1. Figure 5(a) displays the cross-section of the micromirror after completion of the CMOS process. Because the mirror was fabricated from aluminum, which was highly optically reflective (>90%) [16], it could reduce the degree of optical signal loss resulting from transmission. After performing the standard CMOS process, we obtained the final structure of the micromirror by applying a post-processing procedure to remove the sacrificial layer, and to suspend the mirror. The material of sacrificial layer was silicon dioxide. Figure 5(b) presents the mirror after completion of the post-process. First, we cleaned the sample with acetone to remove any impurities. Next, we used an oxide etchant of Silox [18] to remove the sacrificial layer in which the mirror was embedded. In order to avoid the sticking problem, the mirror was immersed in isopropyl alcohol for 20 min and baked in oven at 100 °C for 30 min after the wet etching process.

**Figure 6.**
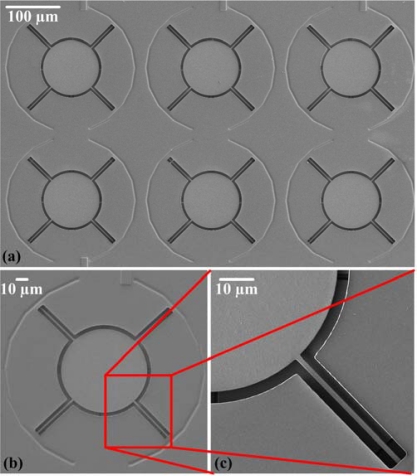
SEM images of (a) the micromirror array, (b) a single mirror, and (c) the beams of the micromirror array, after the post-processing.

**Figure 7.**
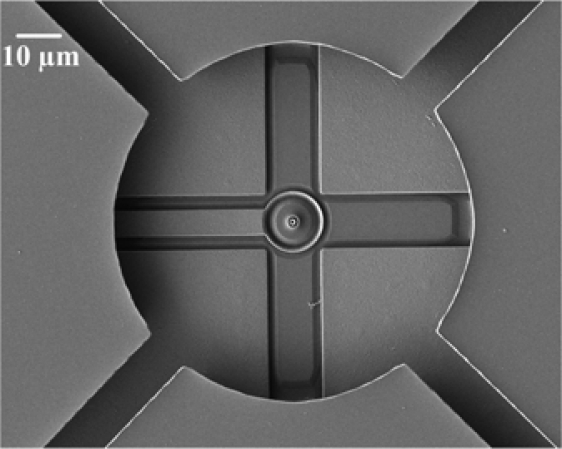
SEM image of the driving electrodes and pin supporter under the micromirror.

Figures 6(a) and (b) shows SEM (scanning electron microscopy) images of the micromirror array and a single mirror, respectively, after completion of the post-process. Figure 6(c) reveals that the sacrificial layer was removed and the micromirror was successfully suspended. Figure 7 displays the fixed electrodes positioned below the suspended mirror and the supportive pin located at the center of the mirror, revealing that they were not damaged during the wet etching process. The etching time control is very important for the undercut problem. A test-key was used to monitor the etch-stop time and to avoid over etching and undercut problems

## 4. Results and Discussion

The profile of the mirror is an important characteristic affecting its performance. We employed a confocal surface optical scan system—a 3D high-resolution, non-contact surface measurement system (Nanofocus) featuring a microscope and a PZT (lead zirconate titanate) actuator—to detect the configuration of the mirror. This system had the ability to calculate the out-of-plane displacement and tilting angle and establish the 3D configuration automatically. The dot pitch is another important parameter affecting the quality of display systems [19]. For a high-resolution display system, the dot pitch is limited to 250 μm.

**Figure 8.**
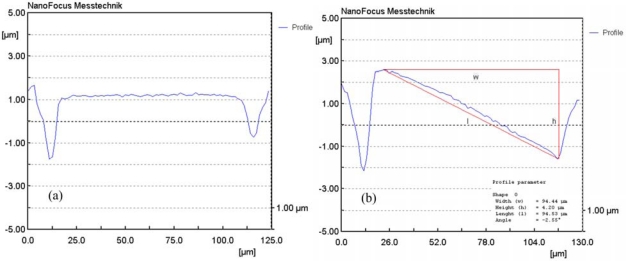
Profile of the micromirror (a) in the absence of a driving voltage and (b) under a driving voltage of 40 V, measured using a confocal surface optical scan system.

**Figure 9.**
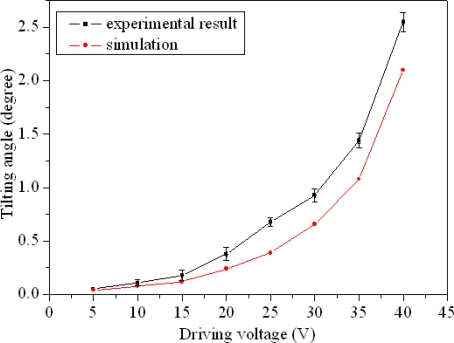
Tilting angles of the mirror at various driving voltages.

Figure 8(a) presents the profile of the mirror in the absence of a driving voltage. The deformation of this mirror was less than 100 nm, i.e., the mirror was flat and not rotated in the initial state. When the driving voltage was less than 15 V, the displacement of the mirror was small; the largest displacement occurred when the driving voltage was 40 V. Figure 8(b) displays the profile of the mirror under a driving voltage of 40 V. The measured result showed that the displacement of the mirror edge was about 2.10 μm at a driving voltage of 40 V, and the tilting angle of the mirror was about 2.55°. Figure 9 reveals the measured tilting angle of the micromirror under various driving voltages. The results depicted that the tilting angle of the mirror was about 0.24° and 0.95° at the driving voltage of 15 V and 30 V, respectively. Furthermore, we employed finite element method (FEM) software (Coventor Ware) to simulate the tilting angle of the micromirror, and the simulated tilting angle of the micromirror under different driving voltages was shown in Figure 9. The simulated results presented that the tilting angle of the mirror was about 0.21° and 0.74° at the driving voltage of 15 V and 30 V, respectively. As shown in Figure 9, the simulated and measured tilting angles for the micromirror were similar under various driving voltages; the slight differences between individual mirrors resulted from variations in the etching during post-processing. The stiffness of the beams decreased because of the post-process etching, leading to that the measured values being larger than the simulated ones.

**Figure 10.**
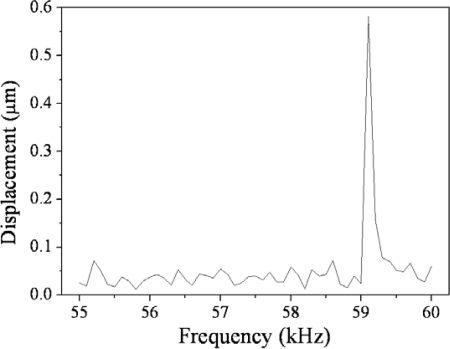
Frequency response of the micromirror.

We employed a MEMS motion analysis system to detect the frequency response of the micromirrors. This measurement system includes a function generator that can apply high-amplitude ac voltages and a microscope to observe the motion of the micromirror. The analyzer takes many images every second to determine the displacement and frequency response of the vibratile object. Figure 10 reveals the frequency response of the micromirror measured by the MEMS motion analysis system. The measured results showed that the resonant frequency of the micromirror was 59.1 kHz.

The vertical axis scan rate is a measure of how many times a display can refresh its whole frame. For video applications, the resolution standards [e.g., SVGA (800 × 600)] require a frame refresh rate of 60 Hz. In this situation, the line rate (horizontal scan) of an SVGA display is 36,000 lines per second. Using a bidirectional-scanning technique, a horizontal scanner operating at 18 kHz requires a scanner retracing rate of 19 – 20 kHz [19]. In future, it is likely that consumers will demand higher resolution and more-fluent video play; therefore, the development of micromirror arrays exhibiting high resonant frequency and small dot pitches will be critical to enable commercially viable displays.

## 5. Conclusions

We have fabricated a micromirror array using the commercial 0.35 μm CMOS process and the post-process. Because this fabrication technique was compatible with the CMOS process, such micromirror arrays have potential to be integrated with circuitry on a chip. The low-cost post-processing method involved simple wet etching with a Silox etchant to etch the sacrificial layer. In the absence of a driving voltage, the micromirror array was very flat (deformation: <100 nm). The mirror was rotatable in four independent directions. The experimental results showed that the tilting angle of the micromirror was about 2.55° under an applied driving voltage of 40 V, and the resonant frequency of the micromirror was 59.1 kHz.

## Figures and Tables

**Table 1.** List of symbols.

**Symbol**	**Description**
*A*	overlapping area of the electrodes
*c*	air damping
*d*	distance of the parallel electrodes
*E*	Young’s modulus of the cantilever beams and the pin
*G*	shear modulus of the torsion bars
*I*	moment of inertia of the cantilever beams
*I_p_*	moment of inertia of the pin
*I_m_*	mass moment of inertia of the mirror
*J*	polar moment of inertia of the torsion bars
*k*	stiffness of spring
*k*_t_	stiffness of torsion spring
*k*_p_	stiffness of pin
*k_e_*	equivalent stiffness of the mirror
*L*	length of the cantilever beams and the torsion bars
*L_p_*	length of the pin
*M_0_*	moment
*m*	mass of the mirror
*r*	radius of the mirror
*V*	driving voltage
*θ*	rotated angle of the mirror
*θ̇*	angular velocity
*θ̈*	angular acceleration
*ε*	permittivity
*β*	stiffness ratio
*ζ*	damping ratio
*ω_n_*	natural frequency of the mirror
*Θ*	amplitude of the response
*ϕ*	phase angle of the response
*r_ω_*	frequency ratio

**Table 2.** Material and geometric properties in the micromirrors.

**Property**	**Value**
*A*	1590 μm^2^
*d*	2.6 μm
*E*	70 GPa
*G*	28 GPa
*I*	2.08 × 10^−26^ m^4^
*I_p_*	3.07 × 10^−27^ m^4^
*J*	3.54 × 10^−25^ m^4^
*k*	0.081 N/m
*k_t_*	1.647 × 10^−10^ N-m/rad
*k_p_*	6.136 × 10^−10^ N-m/rad
*k_e_*	1.35 × 10^−9^ N-m/rad
*L*	60 μm
*L_p_*	1.4 μm
*m*	1.158 × 10^−11^ kg
*r*	50 μm
*V*	0 ∼ 40 V
*ε*	8.85 × 10^−12^ F/m
